# Multiple genetic switches spontaneously modulating bacterial mutability

**DOI:** 10.1186/1471-2148-10-277

**Published:** 2010-09-13

**Authors:** Fang Chen, Wei-Qiao Liu, Abraham Eisenstark, Randal N Johnston, Gui-Rong Liu, Shu-Lin Liu

**Affiliations:** 1 Genomics Research Center (one of The State-Province Key Laboratories of Biomedicine-Pharmaceutics of China), Harbin Medical University, Harbin, China; 2 Department of Microbiology, Peking University Health Science Center, Beijing, China; 3 Department of Microbiology and Infectious Diseases, University of Calgary, Calgary, Canada; 4 Cancer Research Center and University of Missouri, Columbia, Missouri, USA; 5 Department of Biochemistry and Molecular Biology, University of Calgary, Calgary, Canada

## Abstract

**Background:**

All life forms need both high genetic stability to survive as species and a degree of mutability to evolve for adaptation, but little is known about how the organisms balance the two seemingly conflicting aspects of life: genetic stability and mutability. The DNA mismatch repair (MMR) system is essential for maintaining genetic stability and defects in MMR lead to high mutability. Evolution is driven by genetic novelty, such as point mutation and lateral gene transfer, both of which require genetic mutability. However, normally a functional MMR system would strongly inhibit such genomic changes. Our previous work indicated that MMR gene allele conversion between functional and non-functional states through copy number changes of small tandem repeats could occur spontaneously *via *slipped-strand mis-pairing during DNA replication and therefore may play a role of genetic switches to modulate the bacterial mutability at the population level. The open question was: when the conversion from functional to defective MMR is prohibited, will bacteria still be able to evolve by accepting laterally transferred DNA or accumulating mutations?

**Results:**

To prohibit allele conversion, we "locked" the MMR genes through nucleotide replacements. We then scored changes in bacterial mutability and found that *Salmonella *strains with MMR locked at the functional state had significantly decreased mutability. To determine the generalizability of this kind of mutability 'switching' among a wider range of bacteria, we examined the distribution of tandem repeats within MMR genes in over 100 bacterial species and found that multiple genetic switches might exist in these bacteria and may spontaneously modulate bacterial mutability during evolution.

**Conclusions:**

MMR allele conversion through repeats-mediated slipped-strand mis-pairing may function as a spontaneous mechanism to switch between high genetic stability and mutability during bacterial evolution.

## Background

A balance between genetic stability and mutability is essential for bacteria to both retain species identities over long evolutionary times and enable adaptability to changing environments, but little is known about the mechanisms for establishing, maintaining and modulating such a balance. It is richly documented that, under usual growth conditions, genetic stability is largely assured by the functional DNA replication and repair systems. Conversely, when the environment becomes stressful, genetic modifications through mutation or acquisition of exogenous DNA may provide novel traits for greater adaptability of organisms.

Among the known systems for maintaining genetic stability, the DNA mismatch repair (MMR) machinery is the most powerful contributor to the inhibition of mutation and recombination events [1-6]. Bacteria that have elevated mutation rates due to defects in MMR genes, e.g., *mutS, mutL *or *mutH*, are termed mutators or hypermutators and have been isolated from various human pathogens or commensals [7-11]. The mutator genotype may confer a temporary selective advantage for the bacteria under stressful conditions, as it allows for the creation of genetic novelties, including stochastic mutations and incorporation of a great diversity of exogenous DNAs [12,13]. However, as most mutations or intruding DNAs are likely to be harmful, the continuous existence of defective MMR alleles would eventually lead to loss of fitness. One may therefore predict that bacteria should be able to optimize their evolutionary fitness through mechanisms that balance the MMR system between functional and non-functional states, allowing beneficial changes to be made when needed and otherwise minimizing the accumulation of harmful changes. To date, such mechanisms have not been identified.

**Figure 1:**
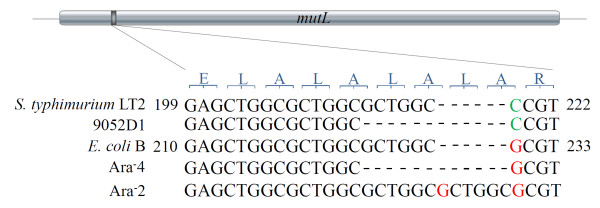
**The defective *mutL *alleles with alternations in the copy number of the 6-bp repeat GCGGGC in populations of *S. typhimurium *(9052D1) and *E. coli *(Ara^-^4 and Ara^-^2)**. The *mutL *sequences of wild type *S. typhimurium *LT2 and *E. coli *B, both containing the three tandem repeats, are shown as references. Also shown is the amino acid sequence of the corresponding section of the deduced MutL protein. The location of the three tandem repeats is shown on the *mutL *gene at the top. The numbering given is for the nucleic acid sequences of *S. typhimurium *LT2 and *E. coli *B.

In previous work, we demonstrated that conversion of *mutL *between functional and defective (6bpΔ*mutL*, which had a deletion of one of three tandem repeats within the gene sequence, Figure 1) alleles may act as a genetic switch to modulate bacterial mutability at the population level [14,15]. Unlike other mechanisms such as the SOS response, the RpoS regulon, DinB error-prone DNA polymerase, RecA, *etc*., which undoubtedly all can generate a high mutability state and can contribute to bacterial evolution [16-23], the repeats-mediated allele conversion via slipped-strand mis-pairing seems to be the only known mechanism that is spontaneous and can respond to environmental changes swiftly. To validate the existence of such a genetic switch(s) and to determine whether mutation or recombination events can actually take place only when the switch is set at the non-functional state, we experimentally locked MMR into either functional or non-functional states so that spontaneous conversion between the two states would become unlikely and then scored bacterial mutability. We found that, when the switch was locked at the functional state, genomic evolutionary events (mutation and recombination) were significantly inhibited. This genetic switch model may be generalized to other bacteria, as small repeats also exist in their MMR genes and may mediate allele conversion through slipped-strand mis-pairing.

## Results

### Detection of conversion between functional and defective *mutL *alleles

**Figure 2:**
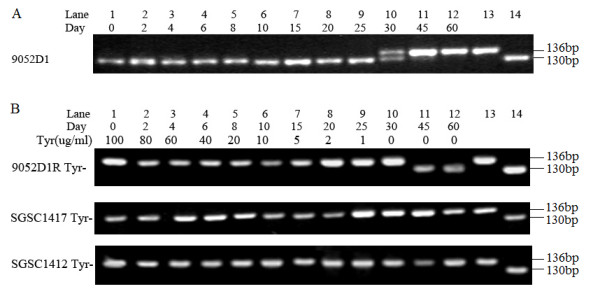
**Detection of allele conversion between *mutL *and 6bpΔ*mutL *by PCR and electrophoresis**. (A) *S. typhimurium *LT7 mutator strain 9052D1, showing a strong tendency for 6bpΔ*mutL *cells to become *mutL *cells once the nutrition stress was removed. (B) Auxotrophic strain 9052D1 Tyr-, showing an obvious tendency for *mutL *cells to become 6bpΔ*mutL *cells soon after inoculation into a stressful environment; SGSC1417 and SGSC1412, no allele conversion detected. Lanes 1-12, PCR products of *mutL *(shown as a 136-bp fragment) or 6bpΔ*mutL *(shown as a 130-bp fragment) alleles in cells of bacterial cultures on days as indicated; lane 13, 136-bp size marker; lane 14, 130 bp size marker.

As the special structure of the tandem GCTGGC repeats in *mutL *is expected to easily lead to deletion or duplication of a copy of the six bases via slipped-strand mis-pairing and in turn to changes in bacterial mutability [14,15], we first needed to experimentally validate the effects of *mutL *allele conversions on mutability. For this, we cultured the *S. typhimurium *LT7 mutator strain 9052D1, which had the 6bpΔ*mutL *genotype [14], and screened for spontaneous allele conversion between *mutL *and 6bpΔ*mutL*. When started, the culture contained only (or mostly) 6bpΔ*mutL *cells. During incubation, the bacterial culture showed a gradual increase in the fraction of wild type *mutL *cells - on day 30, the population contained approximately equal numbers of *mutL *and 6bpΔ*mutL *cells (Figure 2A). A spontaneous *mutL *revertant obtained from 9052D1 on day 60 was saved and designated 9052D1R for subsequent analysis.

To monitor the allele conversion from *mutL *to 6bpΔ*mutL*, we constructed a *tyr *auxotroph in 9052D1R and designated it 9052D1R Tyr-. When inoculated into M9 minimal medium supplemented with gradually decreasing concentrations of tyrosine (nutrients gradually become scarce over time), 6bpΔ*mutL *cells in 9052D1R Tyr- were detected, slowly increased as a fraction of the population and eventually predominated (Figure 2B). Conversely, we did not find any detectable allele conversion in *Salmonella *non-mutators such as SGSC1417 and SGSC1412 (Figure 2B). This may be due either to real absence or to very low frequencies of 6bpΔ*mutL *cells in the bacterial population. We thus increased the screening scale from 100 to over ten thousand single colonies. Nevertheless, no allele conversion was detected either. Detection might become possible had we kept increasing the screening scale, e.g., from thousands to millions of single colonies or more, but such work scales would not be practical by conventional methods. Furthermore, if we did not find the allele conversion even at the million-colony scale, we would still not be able to distinguish between absence and low frequency of 6bpΔ*mutL *cells in these bacteria and, consequently, could not draw any conclusion regarding whether such a spontaneous genetic switch may exist in these bacterial populations. Therefore, alternative methods had to be sought for a definite answer, such as "locking" the *mutL *gene to prevent it from conversion between the wild type and 6bpΔ*mutL *alleles and then inspecting overall mutability changes of the bacterial population.

### Construction of strains containing different *mutL *alleles and evaluation of their mutability

As the inter-conversion between the wild type *mutL *and 6bpΔ*mutL *alleles in *S. typhimurium *LT7 results from copy number changes in the tandem 6 bp repeats (GCTGGC; three copies in wild type *mutL *and two copies in 6bpΔ*mutL*) via slipped-strand mis-pairing [14,24,25], we sought to experimentally prevent the conversion by disrupting the sequence identity between the tandem repeats through base substitution that would not change the amino acids encoded. For example, we "locked" *mutL *allele into its functional state by converting GCTGGC GCTGGC GCTGGC to GCTTGC CCTGGC GCTGGC (modified bases are underlined), using a gene replacement technique [26]. The modified sequence still encodes the amino acids LALALA, but the three 6 bp sequences are no longer identical and thus slipped-strand mis-pairing should be inhibited. We introduced the "locked" *mutL *allele (designated *mutL*^Locked-1^) into *S. typhimurium *LT2 and obtained LT2*^mutL^*L1 (Table 1). Similarly, we "locked" the 6bpΔ*mutL *allele into its non-functional state (designated 6bpΔ*mutL*^Locked^; Table 2) and constructed LT2^6bpΔ*mutL*^L (Table 1). We chose to use *S. typhimurium *LT2 rather than *S. typhimurium *LT7 for this experiment due to the consideration that its whole genome sequence is known so that all genetic manipulations could be made with ease. As a control, we constructed strain LT2*^mutL^*L1U (Table 1), in which the "locked" *mutL *allele was "unlocked" through restoration of the original wild type *mutL *allele sequence (Table 2). As a control experiment to estimate the impact of synonymous substitutions on bacteria, we arbitrarily changed codon 30 in *mutL *from CTG to CTT (both encoding amino acid L) and obtained LT2*^mutL^*LC (Table 1).

**Table 1:** Plasmids and strains used in this study

Plasmid or strain	Genotype and/or description	Reference of source
pGEM-T easy	A-T cloning vector, Ap^R^	Promega
pHSG415	temperature sensitive, Cm^R^, Km^R^, Ap^R^	Reference: [26]
SGSC1417	*S. typhimurium *LT7 non-mutator, wild type	Reference: [41]
SGSC1412	*S. typhimurium *LT2 non-mutator, wild type	Reference: [41]
9052D1	*S. typhimurium *LT7 mutator, genome unchanged, 6bpΔ*mutL*	This study
9052D1R	9052D1, spontaneous *mutL *revertant	This study
LT2*^mutL^*L1	SGSC1412, *mutL *replaced by *mutL*^Locked-1^	This study
LT2*^mutL^*L2	SGSC1412, *mutL *replaced by *mutL*^Locked-2^	This study
LT2*^mutL^*L3	SGSC1412, *mutL *replaced by *mutL*^Locked-3^	This study
LT2*^mutL^*L4	SGSC1412, *mutL *replaced by *mutL*^Locked-4^	This study
LT2*^mutL^*L1U	SGSC1412L1, *mutL*^Locked-1 ^replaced by *mutL*^L1-UL^	This study
LT2*^mutL^*LC	SGSC1412, *mutL *replaced by *mutL*^Locked-C^	This study
LT2^6bpΔ*mutL*^	SGSC1412, *mutL *replaced by 6bpΔ*mutL*	This study
LT2^6bpΔ*mutL*^L	SGSC1412, *mutL *replaced by 6bpΔ*mutL*^Locked^	This study
LT2*^mutS^*L	SGSC1412, *mutS *replaced by *mutS*^Locked^	This study

**Table 2:** DNA repeats within *mutL *and *mutS *genes of *S. typhimurium *LT2 and synonymous substitution of selected bases

Gene	Location	**DNA repeats**^**a**^	Allele	**Substituted bases**^**b**^	Protein	Description
*mutL*	201-218	GCTGGCGCTGGCGCTGGCc	*mutL*^Locked-1^	GCT*T*GC*C*CTGGCGCTGGCc	LALALA	lies in a region that forms a lid over the ATP-binding pocket of MutL protein
			*mutL*^L1-UL^	GCT*G*GC*G*CTGGCGCTGGCc		
	190-195	AAAAAA	*mutL*^Locked-2^	AA*G*AAA	KK	lies in a relatively disordered structure of ATP-binding pocket of MutL protein
	491-496	gAAAAAA	*mutL*^Locked-3^	gA*G*AA*G*A	EK	lies in α helic E of MutL protein
	735-743	CACCACCACg	*mutL*^Locked-4^	CAC*G*AC*T*ACg	TTT	lies in a relatively disordered structure of MutL C-terminal demonization domain
	82-90	AAAGAGCTG	*mutL*^Locked-C^	AAAGAGCT*T*	KEL	lies in the α helic A of MutL protein
6bpΔ*mutL*	201-212	GCTGGCGCTGGc	6bpΔ*mutL*^Locked^	GCT*T*GC*G*CTGGCc	LALA	lies in a region that forms a lid over the ATP-binding pocket of MutL protein
*mutS*	1189-1195	AAAAAAA	*mutS*^Locked^	AA*G*AAAA	KK	lies in the C-terminal end of helic α16, which forms the core domain of the MutS protein

a, The first codon encoding the protein is underlined.

b, Italic characters represent modified bases; characters in small case indicate neighboring base of the sequence.

**Figure 3:**
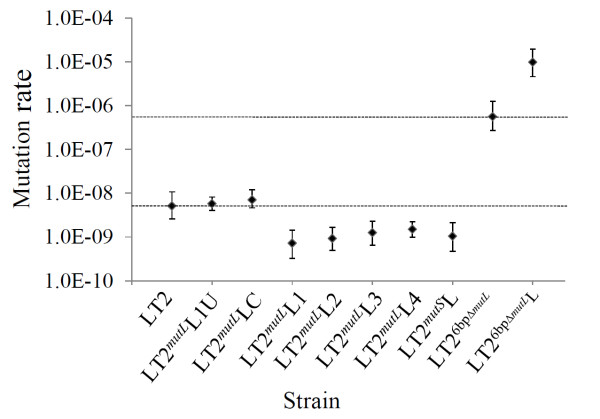
**Mutation rates to rifampicin resistance determined by fluctuation tests in isogenic strains carrying different *mutL *alleles**. Error bars indicate 95% confidence limits. For visual comparison, the dotted horizontal lines illustrate the mutation rates of LT2 (lower line) and LT2^6bpΔ*mutL *^(upper line), respectively.

Mutation rates of these constructed isogenic strains shown in Figure 3 unambiguously demonstrated the key roles of *mutL *allele conversion in modulating genetic stability of a bacterial population - when *mutL *was locked into the functional state (strain LT2*^mutL^*L1), the mutability was obviously suppressed compared to its wild type ancestor (LT2), whereas when *mutL *was locked into its defective state (strain LT2^6bpΔ*mutL*^L), the mutability was significantly increased compared to its parent strain (LT2^6bpΔ*mutL*^). Moreover, the mutability of the wild type strain level was seen again when the locked *mutL *allele was restored to its convertible wild type sequence. The synonymous base substitution process itself did not lead to apparent influences to the bacterial mutability, as strain LT2*^mutL^*LC exhibited a mutation rate similar to that of wild type LT2 (Figure 3).

**Figure 4:**
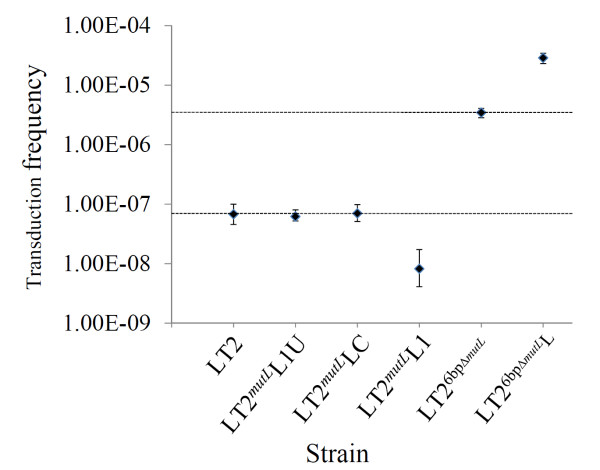
**Frequencies of transduction of the *leu *gene, with *S. typhi *Ty2 as the donor, in isogenic strains carrying different *mutL *alleles**. Error bars represent the standard deviation. For visual comparison, the dotted horizontal lines illustrate the mutation rates of LT2 (lower line) and LT2^6bpΔ*mutL *^(upper line), respectively.

We also examined changes in genetic stability by comparing recombination frequencies among these bacterial strains and found the same trend (Figure 4). In conclusion, the prevention of conversion of *mutL *from the functional to a defective state significantly reduced the capacity of the bacteria to accept genetic novelties that would facilitate their adaptation to environmental pressures, *e.g*., through mutation to develop antibiotic resistance (Figure 3) or through recombination to acquire needed biosynthesis genes, even when the genes were available in the environment (Figure 4).

### Other DNA repeats within *mutL *and *mutS *genes in *S. typhimurium *LT2

The fact that mutability of a bacterial population comprised of LT2*^mutL^*L1 was reduced but not completely abolished prompted us to search for other sequence repeats in *mutL *as well as other MMR genes that might also play the roles of genetic switches through internal tandem repeat copy number changes. As a result of this more extensive search, we found three additional candidate DNA repeats in *mutL *and one in *mutS *in *S. typhimurium *LT2 (Table 2), all of which could possibly mediate additions or deletions of nucleotides *via *slipped-strand mis-pairing during replication and thus might influence genetic stability/mutability. To evaluate their potential roles as genetic switches, we constructed a series of isogenic strains, including LT2*^mutL^*L2, LT2*^mutL^*L3, LT2*^mutL^*L4 and LT2*^mutS^*L (Table 1), in which at least one base in the repeats within *mutL *or *mutS *was changed to disrupt the sequence identity among the repeats. As shown in Figure 3, the range of mutability in the bacterial populations decreased 4-7 fold compared to that of the wild type LT2 strain, indicating that these repeats may also serve as switches in modulating the bacterial genetic stability/mutability. We are initiating a series of experiments to lock all of these potential convertible sites in *mutL *and *mutS *at the functional states in the same bacterial cell to determine whether the mutability of the bacterial population started with this genetically manipulated cell might be further reduced or even abolished.

### DNA repeats within MMR genes in other bacterial species

**Figure 5:**
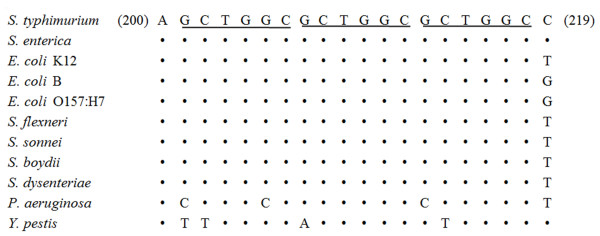
**Alignment of the three 6 bp tandem repeats within *mutL *of *S. typhimurium *LT2 with homologous sequences of other bacteria**. Positions at which the sequences of other bacteria are identical to those of *S. typhimurium *LT2 are indicated by dots. The numbering given in brackets is for the *S. typhimurium *LT2 nucleic acid sequence.

We wondered whether the *mutL*-6bpΔ*mutL *switch model that we describe in *Salmonella *might be applicable to other species, as all bacteria should experience a similar need to modulate genetic stability and mutability. After a careful examination of multiple bacterial genomes, we found that the exact 6 bp tandem repeats that are present in the *mutL *gene of *S. typhimurium *were identifiable only in bacteria that are very closely related to *Salmonella*, such as *Escherichia *and *Shigella *(Figure 5). In more distantly related bacteria, such as *Yersinia *or *Pseudomonas*, there were nucleotide differences in that particular site (Figure 5) that were sufficient to abolish the sequence identity required for allele conversion by slipped-strand mis-pairing. Nevertheless, we found numerous other short DNA repeats (mostly mono- and tri-nucleotide repeats) in other regions of the *mutL *or *mutS *genes in over 100 bacterial species (representative examples are shown in Additional file 1: Supplemental Table S1), raising the possibility that genetic switches may be common in a wide range of bacterial species. It is of great interest to notice that in almost all of the common bacterial pathogens several repeats were identified in *mutL *and *mutS *genes (Additional file 1: Supplemental Table S2). This may enable the pathogenic organisms to counteract host antibacterial responses by allowing rapid evolutionary bursts mediated *via *transient MMR defects.

## Discussion

The concept of genetic switches for spontaneously modulating mutability is important, as it reconciles the two seemingly conflicting requirements of genetic stability and mutability. Without genetic stability, species continuity would not exist; without mutability, organisms would hardly be able to adapt to changing environments by generating genetic novelty.

The DNA mismatch repair system is well known for its role in maintaining genetic stability [1-3,5,6,12,27-32]. Considerable effort has been devoted to the elucidation of the emergence and molecular causes of MMR defects and their impacts on evolution. However, the roles of variable MMR gene function in balancing genetic stability and mutability have received little attention. In this study, we experimentally validated our previous observations about the consequences of *mutL*/6bpΔ*mutL *allele conversion in bacterial genomic stability and further demonstrated that such conversion might occur in multiple MMR genes, serving as spontaneous genetic switches that bacteria can use to modulate mutability at the population level during evolution.

Some of the results seemed inconsistent with the genetic switch hypothesis. For example, although we readily detected *mutL *to 6bpΔ*mutL *conversion in *S. typhimurium *LT7 mutators, we did not find detectable allele conversion in *Salmonella *non-mutators (*e.g*., SGSC1417 and SGSC1412; see Figure 2B), even though our hypothesis would predict the rare appearance of a 6bpΔ*mutL *genotype under these experimental conditions. However, the "negative findings" do not necessarily mean that the *mutL *to 6bpΔ*mutL *conversion was really negative. For instance, if the 6bpΔ*mutL *frequency was 10^-8 ^at the start of the experiment and was increased to 10^-4 ^at the end of the experiment, this ten thousand-fold amplification would still be well beyond the detection capability by the available techniques. We thus attempted to tackle this problem from the opposite direction: if we prevented the *mutL *to 6bpΔ*mutL *conversion when the 6bpΔ*mutL *genotype was predicted as necessary in a challenging growth environment, would adaptability of the modified bacteria be reduced compared to the wild type controls? When *mutL *was prevented from conversion to 6bpΔ*mutL *by nucleotide replacement, we found that bacterial mutability as evidenced by adaptability in challenging environments was significantly reduced. This result demonstrated that conversion from *mutL *to 6bpΔ*mutL *plays important roles in bacterial adaptation; success to detect the 6bpΔ*mutL *at high frequency in some but not all bacterial strains in the experiments may just reflect genetic variations among them, which however will not negate the fact that conversion from *mutL *to 6bpΔ*mutL *may render the bacteria greater adaptability in challenging environments.

We emphasize here that the genetic switch works at the population level. Specifically, we postulate that 6bpΔ*mutL *cells pre-exist as rare variants in bacterial populations, rather than arising in response to environmental or metabolic challenge. One key point here in the postulation is that they do exist, no matter how low their frequencies might be. Under normal conditions, 6bpΔ*mutL *cells would not impose any harmful effects because of their low frequencies. Once under stress, bacteria may require novel biological traits to adapt and survive. By chance, some rare 6bpΔ*mutL *cells in the population may have accumulated "beneficial" nucleotide changes or acquired "useful" exogenous genes and thus will be selected to propagate to increasing subpopulation sizes, eventually predominating in the population. We envision that, following successful adaptation, the 6bpΔ*mutL *cells would provide no further benefits or may even facilitate deleterious genomic changes and would consequently become once again rare in the bacterial populations. In this way, the *mutL*-6bpΔ*mutL *switch may establish and maintain a dynamic balance between genetic stability and mutability under different environmental conditions.

Previous work with evolving *E. coli *populations has also demonstrated spontaneously arising *mutL *mutators as the result of changes in repeat length [33]. The reported repeat unit in that case was the 6-bp string, CTGGCG, beginning at position 213 of the *mutL *gene in *E. coli *B. However, we believe that the variable tandem repeat unit should be identified as GCTGGC, starting at position 212 of the *mutL *gene in *E. coli *and also having three repeats in functional *mutL*, exactly as what we have found in *S. typhimurium*. Although *E. coli *B has the two overlapping sets of tandem repeats, we note that three copies of GCTGGC, rather than those of CTGGCG, are conserved throughout the *Salmonella*-*E. coli*-*Shigella *complex (Figure 5) and thus are more likely to function as a common mechanism for modulating genetic stability/mutability in these bacteria.

Although in this study we primarily focused on the *mutL*-6bpΔ*mutL *switch, our bioinformatic analysis also predicted four other sets of DNA repeats in MMR genes of *S. typhimurium *LT2, with three in *mutL *and one in *mutS*. Because locking these repeats into their functional states also yielded a significant decrease in mutability in the bacterial populations (Figure 3), we suggest that these repeats may also function as genetic switches. It is likely that these multiple *mutL *or *mutS *switches work stochastically to spontaneously modulate genetic stability/mutability in bacterial populations, although it is possible that some of the switches might be more functionally important than others according to the ease with which conversion between functional and defective alleles occurs and the effect of the defect on the protein encoded by the MMR gene [15].

**Figure 6:**
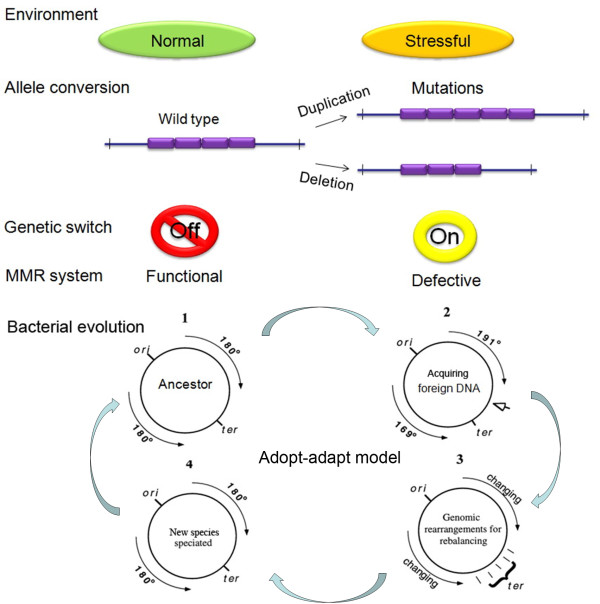
**A refined Adopt-Adapt Model of bacterial speciation with the spontaneous genetic switch component**.

Based on the findings reported in this study, we have updated the adopt-adapt model of bacterial evolution [34,35] by assigning a key role for repeats-mediated allele conversion of MMR genes (Figure 6). We suggest first that foreign DNA (acquired by transduction, conjugation or transformation) will be most readily incorporated into the genome of the recipient cell when MMR function is diminished. Second, if the incoming DNA segment is large (100 kb or greater), its incorporation at some site within the genome would cause the normally balanced *oriC *to *terC *distances to become unequal in the two replicores. As bacteria with unbalanced genomes are less fit and show increased generation times [36], the bacteria that have incorporated the additional DNA ('adopt') may undergo further genomic rearrangements ('adapt') mediated by recombination among partially homologous sequences and facilitated by defective MMR activity to restore *oriC *to *terC *balance. We further speculate that bacterial speciation events, such as the recent divergence between *Salmonella choleraesuis *and *S. paratyphi *C [37], may arise in part through the rapid accumulation of non-synonymous mutations that are also attributable to variable genetic mutability switches (though not necessarily the *mutL*-6bpΔ*mutL *switch).

It is worth noting that over-representation of small DNA repeats have also been identified in other stress response genes and virulence genes in bacteria [38-40], but none has so far been directly implicated in a switch-like role for mutability-modulation during bacterial adaptation to environmental challenges. The concept of spontaneous genetic switches based on repeats-mediated allele conversion may therefore be a useful starting point for further investigation of regulatory mechanisms for bacterial adaptive behavior and evolution.

## Conclusions

MMR allele conversion through repeats-mediated slipped-strand mis-pairing could work as a mechanism for spontaneous switching between states of high genetic stability and mutability during bacterial evolution.

## Methods

### Bacterial strains, plasmids and media

Laboratory strains and plasmids used in this study are listed in Table 1. *S. typhimurium *LT7 mutant 9052D1 was the first strain to have the MMR genes sequenced and was found to have the 6bpΔ*mutL *genotype [14]. *S. typhimurium *LT7 non-mutator and *S. typhimurium *LT2 were originally isolated by Lilleengen [41] in the 1940s as representative strains of phage types LT1 through LT22. Bacterial strains were routinely grown in Luria Bertani (LB) broth as liquid media or on agar plates at 37°C.

### Experimental selection systems for allele conversion

*S. typhimurium *LT7 mutator 9052D1 was propagated at 37°C by 100-fold dilutions every 12 hours into 5 ml of fresh LB broth for 60 days (~1,000 generations). Samples from each population were frozen on days 0, 2, 4, 6, 8, 10, 15, 20, 25, 30, 45, and 60 to monitor the spontaneous allele conversion from 6bpΔ*mutL *to *mutL*. Spontaneous *mutL *revertants obtained from the above experiments were designated as 9052D1R. Auxotrophic mutants of 9052D1R, SGSC1417 and SGSC1412 were constructed by Tn*10 *insertion inactivation in biosynthetic genes, e.g., in *tyrA*, as described previously [14]. The auxotrophs, designated as 9052D1R Tyr-, SGSC1417 Tyr- and SGSC1412 Tyr-, respectively, were grown at 37°C by 100-fold dilutions every 12 hours into 5 ml of fresh M9 minimal medium supplemented with gradually decreasing concentrations of tyrosine from 100 μg/ml to 0 μg/ml. Trace amounts of the nutrient were used to sustain bacterial growth for some time in order for mutations to accumulate to circumvent the genetic defect in amino acid synthesis. Samples from each population were frozen on days 0, 2, 4, 6, 8, 10, 15, 20, 25, 30, 45, and 60 to monitor the spontaneous allele conversion from *mutL *to 6bpΔ*mutL*.

### Detection of *mutL *and 6bpΔ*mutL *alleles

Polymerase chain reaction (PCR) was used for detection of bacterial cells carrying the *mutL *or 6bpΔ*mutL *allele, with the primers L-F1 and L-R1 listed in Additional file 1: Supplemental Table S3. The PCR products, a 130-bp fragment on 6bpΔ*mutL *and a 136-bp fragment on *mutL*, were resolved by agarose gel electrophoresis with 5% agarose (Amersco SFRTM), at 5 V/cm, for 3 h. The gel was photographed with the Bio-Rad Gel Doc system (Bio-Rad) following electrophoresis.

### Gene locking and unlocking

Synonymous substitutions were introduced into the DNA repeats in *mutL *or *mutS *by PCR-based site-directed mutagenesis to construct the locked or unlocked alleles (Table 2), with primers listed in Additional file 1: Supplemental Table S3. The genomic sequence of *Salmonella typhimurium *LT2 was used as templates for the design of primers. Overlap extension PCR was carried out in two stages. The first stage consisted of a set of two PCR reactions: one from an upstream flanking primer (F1) to the negative-sense mutagenesis primer (R1) and the other from a downstream flanking primer (F2) to the positive-sense mutagenesis primer (R2). In the second stage, PCR products from the first stage were used as the templates for a PCR reaction using only the flanking primers (F1, R2). PCR products were purified from agarose gels using AxyPrep DNA gel extraction kits (Axygen) and an A-tailing nucleotide was added with Taq DNA polymerase (Promega) prior to cloning into pGEM T-easy vector (Promega) and transformation of chemically prepared competent *E. coli *DH5α cells. Transformants were selected on LB agar plates with 100 μg/ml ampicillin. Genes cloned in the resulting plasmids were sequenced by Shanghai Sangon Biological Engineering Technology & Services Co., Ltd. for both strands.

### Gene replacement

*mutL *or *mutS *sequences were obtained from BamHI and EcoRI-digested pGEM-T easy plasmids containing locked or unlocked alleles and subcloned into BamHI and EcoRI-digested pHSG415, a temperature-sensitive vector used for allele replacement via homologous recombination [26]. The recombinant pHSG415 plasmids (pHSG415 containing locked or unlocked alleles) were first transformed into chemically prepared competent *E. coli *DHα cells, and then purified and transformed via electroporation (Bio-Rad) into LT2 strains. The allelic exchange experiments were carried out as described by White [26]. Briefly, bacterial strains containing the recombinant plasmids were grown at 42°C by 100-fold dilutions into 5 ml LB broth with 100 μg/ml ampicillin (LB/Amp broth) daily for 4 days. Dilutions of the final cultures were plated on LB/Amp plates and grown overnight at 42°C to select Amp-resistant colonies; 5-8 cointegrate colonies thus obtained were grown at 28°C by 100-fold dilutions into 5 ml LB broth daily for 4 days. Dilutions of the final cultures were plated on LB plates and incubated overnight at 28°C and then replica-plated onto LB and LB/Amp plates to select Amp-sensitive colonies. Strains with successful allele-replacement were confirmed by sequencing.

### Mutation rate measurements

Fluctuation tests [42,43] were conducted to determine the mutation rates of *Salmonella *strains carrying different *mutL *alleles. At least 30 cultures of a given strain were grown from inocula of approximately 1,000 cells for each fluctuation test. Cultures were grown to stationary phase before selective plating on LB agar plates containing 100 μg/ml rifampicin (Rif). Final population sizes were estimated by growing and sampling three extra cultures taken at random for each strain and measuring the total number of colony forming units (CFU) in LB plates without rifampicin. The mutation rate determinations and the statistical analysis from the fluctuation assays were carried out using the MSS Maximum-Likelihood Method as described previously [43,44].

### Recombination frequencies estimated by transduction

Bacteriophage P22-mediated transduction was used to inactivate *leu, metC *or *proB *in *S. typhimurium *LT2 or its isogenic strains by transferring Tn*10 *insertions as previously described [45,46]. For each transduction, 100 μl of recipient cells grown to 5 × 10^8 ^CFU/ml were infected with 10 μl of phage lysate diluted to yield a phage:bacteria ratio of 1:10. Bacterial cultures and phage lysates were mixed directly on M9 minimal medium plates containing glucose (8 mg/ml) and incubated at 37°C for 18 h. The transduction frequency was calculated by determining the number of cells growing on M9 plates divided by the total number of colonies on the LB plates. All experiments were performed in triplicate, and the mean value was recorded.

### Bioinformatics analysis of DNA repeats in *mutL *and *mutS *genes

The complete bacterial genome sequences were downloaded from Entrez Genomes (http://www.ncbi.nlm.nih.gov). We screened DNA repeats with motifs of 1-6 nt in length within *mutL *and *mutS *genes in over 100 bacterial species. To reduce bias resulting from some genera being represented by multiple species, we counted only one species per genus in each collection. The definition of DNA repeats is: for mononucleotide runs, ≥6 nt (six motifs); for dinucleotide runs, ≥10 nt (five motifs); for trinucleotide runs, ≥9 nt (three motifs); for tetranucleotide runs, ≥12 nt (three motifs); for pentanucleotide runs, ≥15 nt (three motifs); and for hexanucleotide runs, ≥18 nt (three motifs).

### Funding

This work was supported by a Canadian Institutes of Health Research grant to RNJ; a grant of National Natural Science Foundation of China (NSFC30970078) and a grant of Natural Science Foundation of Heilongjiang Province of China to GRL; a grant from Harbin Medical University, a 985 Project grant of Peking University Health Science Center, grants of National Natural Science Foundation of China (NSFC30870098, 30970119, 81030029) and Specialized Research Fund for the Doctoral Program of Higher Education (SRFDP, 20092307110001) to SLL.

## Authors' contributions

FC carried out the experiments and wrote the first draft of the manuscript. WQL discovered the 6 bp deletion in *mutL *of LT7 mutator. GRL initiated the *mutL *project with physical mapping. AE provided the LT7 bacterial strains and mutants. RNJ contributed reagents and helped with editing the manuscript. SLL raised the original research question, designed the project, supervised the experimental work and analyses, and wrote the final version of the manuscript. All authors read and approved the final manuscript.

## Supplementary Material

Additional file 1 **Supplemental Materials**. The file contains three supplemental tables, i.e., Supplemental Table S1, 2 and 3. Click here for file
