# Supplementary material for: Multiple genetic switches spontaneously modulating bacterial mutability
Source: BMC Evol Biol. 2010 Sep 13;10:277. doi: 10.1186/1471-2148-10-277 (PMC2955026; doi:10.1186/1471-2148-10-277)
Supplement: Additional file 1 — Supplemental Materials. The file contains three supplemental tables, i.e., Supplemental Table S1, 2 and 3. [file 1471-2148-10-277-S1.DOC]

**Supplemental Materials**

**Table S1. Examples of bacteria with DNA repeats in *mutL* and *mutS* genes.**

| Bacteria | No. of repeats | |
| --- | --- | --- |
|  | *mutL* | *mutS* |
| *Bacillus cereus* 10987 | 9 | 10 |
| *Buchnera aphidicola* Sg | 19 | 27 |
| *Campylobacter jejuni* NCTC 11168 | ∕ | 18 |
| *Clostridium acetobutylicum* ATCC824 | 5 | 12 |
| *Clostridium tetani* E88 | 8 | 14 |
| *Francisella tularensis subsp. tularensis* FSC 198 | 6 | 16 |
| *Lactococcus lactis* subsp. lactis IL1403 | 9 | 10 |
| *Prochlorococcus marinus* MIT 9312 | ∕ | 20 |
| *Staphylococcus aureus* RF122 | 9 | 8 |
| *Streptococcus mutans* UA159 | 7 | 12 |
| *Thermoanaerobacter tengcongensis* MB4 | 8 | 11 |
| *Thermus thermophilus* HB27 | 10 | 10 |

**Table S2. DNA repeats within *mutL* and *mutS* genes of common bacterial pathogens**

| Bacterial pathogen | *mutL* | |  | *mutS* | |
| --- | --- | --- | --- | --- | --- |
|  | Repeats | Location |  | Repeats | Location |
| *Bacillus anthracis* Ames | (AGA)3 | 195-203 |  | (A)6 | 821-826 |
|  | (TTA)3 | 368-376 |  | (A)6 | 1529-1534 |
|  | (A)6 | 401-406 |  | (GAA)3 | 1663-1671 |
|  | (A)7 | 976-982 |  | (A)6 | 2010-2015 |
|  | (A)6 | 1018-1023 |  | (GAA)3 | 2494-2502 |
|  | (A)7 | 1662-1668 |  | (A)6 | 2539-2544 |
|  | (A)6 | 1678-1683 |  | (A)7 | 2596-2602 |
|  | (ACA)3 | 1786-1794 |  | (A)7 | 2657-2663 |
| *Bordetella parapertussis* 12822 | (GC)5 | 125-134 |  | (GC)5 | 641-650 |
|  | (GGC)3 | 154-162 |  | (CG)5 | 1987-1996 |
|  | (GCC)3 | 1057-1065 |  | (GC)5 | 731-740 |
|  |  |  |  | (CG)5 | 2077-2086 |
|  |  |  |  | (CCG)3 | 314-322 |
|  |  |  |  | (CCG)3 | 404-412 |
| *Bordetella pertussis* Tohama I | (GC)5 | 125-134 |  | (GC)5 | 641-650 |
|  | (GGC)3 | 153-162 |  | (CG)5 | 1987-1996 |
|  | (GCC)3 | 1057-1065 |  | (CCG)3 | 314-322 |
| *Burkholderia mallei* ATCC 23344 | (CG)5 | 1800-1809 |  | (AGC)3 | 30-38 |
|  | (GGA)3 | 1683-1691 |  | (C)7 | 1043-1049 |
|  |  |  |  | (GCC)3 | 1257-1265 |
|  |  |  |  | (CCG)3 | 1530-1538 |
|  |  |  |  | (C)6 | 2533-2538 |
|  |  |  |  | (CCG)3 | 2537-2545 |
| *Burkholderia pseudomallei* K96243 | (GGA)3 | 1683-1691 |  | (AGC)3 | 30-38 |
|  | (CG)5 | 1800-1809 |  | (C)7 | 1043-1049 |
|  |  |  |  | (GCC)3 | 1257-1265 |
|  |  |  |  | (CCG)3 | 1530-1538 |
|  |  |  |  | (C)6 | 2533-2538 |
| *Campylobacter jejuni* NCTC 11168 | / | / |  | (T)7 | 95-101 |
|  |  |  |  | (A)8 | 279-286 |
|  |  |  |  | (A)6 | 435-440 |
|  |  |  |  | (A)6 | 656-661 |
|  |  |  |  | (T)6 | 714-719 |
|  |  |  |  | (A)6 | 723-728 |
|  |  |  |  | (T)6 | 735-740 |
|  |  |  |  | (A)7 | 802-808 |
|  |  |  |  | (A)6 | 879-885 |
|  |  |  |  | (A)6 | 910-915 |
|  |  |  |  | (A)6 | 1552-1557 |
|  |  |  |  | (A)7 | 1604-1610 |
|  |  |  |  | (A)7 | 1678-1684 |
|  |  |  |  | (A)6 | 1724-1729 |
|  |  |  |  | (A)6 | 1814-1819 |
|  |  |  |  | (A)7 | 1906-1912 |
|  |  |  |  | (T)6 | 1139-1144 |
|  |  |  |  | (ATC)3 | 1258-1266 |
| *Clostridium perfringens* 13 | (A)7 | 580-586 |  | (T)6 | 67-72 |
|  | (A)6 | 777-782 |  | (A)6 | 167-172 |
|  | (AGA)3 | 1011-1020 |  | (A)6 | 363-368 |
|  | (A)6 | 1025-1030 |  | (A)6 | 725-730 |
|  | (AAG)3 | 1043-1051 |  | (A)6 | 836-841 |
|  | (A)9 | 1132-1140 |  | (A)6 | 2046-2051 |
|  | (A)6 | 1323-1328 |  | (AAG)3 | 2537-2545 |
| *Clostridium tetani* E88 | (A)6 | 141-146 |  | (T)6 | 67-72 |
|  | (T)6 | 260-265 |  | (T)6 | 97-102 |
|  | (ATC)3 | 486-494 |  | (A)6 | 363-368 |
|  | (T)6 | 847-852 |  | (TTA)3 | 514-522 |
|  | (A)7 | 643-649 |  | (A)6 | 548-563 |
|  | (T)6 | 822-827 |  | (A)6 | 716-721 |
|  | (A)6 | 1382-1387 |  | (A)6 | 932-937 |
|  | (A)6 | 1840-1845 |  | (A)6 | 1089-1094 |
|  |  |  |  | (A)6 | 1260-1265 |
|  |  |  |  | (A)6 | 1754-1759 |
|  |  |  |  | (A)6 | 2226-2231 |
|  |  |  |  | (A)6 | 2461-2466 |
|  |  |  |  | (GAA)3 | 2527-2535 |
|  |  |  |  | (A)7 | 2636-2642 |
| *Escherichia coli* K12 | (A)6 | 190-195 |  | (CGC)3 | 2406-2414 |
|  | (GCTGGC)3 | 201-218 |  |  |  |
| *Haemophilus ducreyi* 35000HP | (ACA)3 | 633-641 |  | (T)6 | 110-115 |
|  | (A)7 | 1380-1386 |  | (A)6 | 127-132 |
|  | (A)6 | 1423-1428 |  | (G)6 | 167-172 |
|  | (T)7 | 1656-1662 |  | (T)7 | 1799-1805 |
|  |  |  |  | (A)6 | 2468-2473 |
|  |  |  |  | (A)7 | 2577-2583 |
| *Haemophilus influenzae* KW20 | (T)7 | 449-455 |  | (A)6 | 133-138 |
|  | (A)7 | 1585-1591 |  | (A)6 | 1157-1162 |
|  | (TCA)3 | 1714-1722 |  | (TTA)3 | 1606-1614 |
|  |  |  |  | (A)7 | 2155-2161 |
|  |  |  |  | (A)7 | 2396-2402 |
| *Helicobacter pylori* 26695 | / | / |  | (AAT)4 | 46-57 |
|  |  |  |  | (T)6 | 121-126 |
|  |  |  |  | (T)7 | 399-405 |
|  |  |  |  | (TGA)3 | 426-431 |
|  |  |  |  | (A)6 | 499-504 |
|  |  |  |  | (A)6 | 695-700 |
|  |  |  |  | (A)6 | 707-712 |
|  |  |  |  | (T)6 | 825-830 |
|  |  |  |  | (A)9 | 902-910 |
|  |  |  |  | (T)6 | 1050-1055 |
|  |  |  |  | (T)6 | 1431-1436 |
|  |  |  |  | (T)6 | 1497-1052 |
|  |  |  |  | (A)6 | 1693-1698 |
|  |  |  |  | (A)7 | 1981-1987 |
|  |  |  |  | (T)6 | 2112-2117 |
|  |  |  |  | (A)6 | 2208-2213 |
| *Legionella pneumophila* Lens | (A)6 | 483-488 |  | (T)6 | 103-108 |
|  | (C)6 | 544-549 |  | (A)7 | 237-243 |
|  | (T)6 | 587-592 |  | (A)6 | 407-412 |
|  | (T)7 | 957-963 |  | (TGG)3 | 834-842 |
|  | (ATC)3 | 1149-1157 |  | (A)7 | 1349-1355 |
|  | (A)7 | 1177-1183 |  | (A)6 | 1473-1478 |
|  | (T)7 | 1518-1524 |  | (A)8 | 2448-2455 |
|  | (T)7 | 1545-1551 |  |  |  |
| *Neisseria gonorrhoeae* FA1090 | (GGC)3 | 145-153 |  | (A)6 | 406-411 |
|  | (CGG)3 | 180-188 |  | (A)7 | 844-850 |
|  | (CCG)3 | 404-412 |  | (GCC)3 | 1624-1632 |
|  | (A)7 | 1261-1267 |  | (A)7 | 2129-2135 |
|  |  |  |  | (CGC)3 | 2196-2204 |
| *Neisseria meningitidis* MC58 | (GGC)3 | 145-153 |  | (A)6 | 406-411 |
|  | (CGG)3 | 180-188 |  | (A)6 | 844-849 |
|  | (CCG)3 | 404-412 |  | (GCC)3 | 1624-1632 |
|  | (A)7 | 1261-1267 |  | (A)7 | 2129-2135 |
|  | (CGC)3 | 1479-1487 |  | (CGC)3 | 2198-2206 |
|  | (A)6 | 1505-1510 |  |  |  |
| *Pseudomounas aeruginosa* PA01 | / | / |  | (CCA)3 | 1178-1186 |
| *Salmonella typhi* CT18 | (A)6 | 190-195 |  | (A)7 | 1189-1195 |
|  | (GCTGGC)3 | 201-218 |  |  |  |
|  | (A)6 | 491-496 |  |  |  |
|  | (CAC)3 | 735-743 |  |  |  |
|  | (CGA)3 | 1253-1261 |  |  |  |
| *Shigella boydii* | (A)6 | 190-195 |  | (CGC)3 | 2370-2378 |
|  | (GCTGGC)3 | 201-218 |  |  |  |
| *Staphylococcus aureus* RF122 | (A)6 | 415-420 |  | (T)6 | 70-75 |
|  | (A)8 | 1021-1028 |  | (A)6 | 163-168 |
|  | (A)6 | 1055-1060 |  | (A)6 | 805-810 |
|  | (TGA)3 | 1227-1235 |  | (A)6 | 1272-1277 |
|  | (ATA)3 | 1247-1255 |  | (A)6 | 1567-1572 |
|  | (A)6 | 1282-1287 |  | (ACA)3 | 1587-1595 |
|  | (A)6 | 1792-1797 |  | (A)6 | 2190-2195 |
|  | (A)6 | 1807-1812 |  | (A)6 | 2368-2373 |
|  | (A)6 | 1846-1851 |  |  |  |
| *Staphylococcus haemolyticus* JCSC1435 | (A)6 | 415-420 |  | (T)6 | 100-105 |
|  | (A)8 | 1021-1028 |  | (A)6 | 163-168 |
|  | (A)7 | 1157-1163 |  | (A)6 | 805-810 |
|  | (ATA)3 | 1214-1222 |  |  |  |
|  | (CAT)3 | 1292-1300 |  |  |  |
|  | (A)6 | 1574-1579 |  |  |  |
|  | (A)6 | 1810-1815 |  |  |  |
|  | (A)7 | 1825-1831 |  |  |  |
|  | (A)6 | 1864-1869 |  |  |  |
|  | (A)6 | 1893-1898 |  |  |  |
| *Streptococcus agalactiae* NEM316 | (T)6 | 1111-116 |  | (T)6 | 69-74 |
|  | (A)8 | 654-661 |  | (ATG)3 | 110-118 |
|  | (A)6 | 1197-1202 |  | (A)6 | 286-291 |
|  | (T)6 | 1375-1380 |  | (A)7 | 548-554 |
|  | (A)6 | 1735-1740 |  | (T)7 | 957-963 |
|  | (A)6 | 1574-1579 |  | (A)6 | 1369-1374 |
|  | (AAG)3 | 1663-1671 |  | (T)6 | 1428-1433 |
|  |  |  |  | (A)6 | 1449-1454 |
| *Streptococcus mutans* UA159 | (A)7 | 157-163 |  | (A)6 | 49-54 |
|  | (A)6 | 244-249 |  | (T)6 | 69-74 |
|  | (AAG)3 | 398-406 |  | (ATG)3 | 110-118 |
|  | (A)7 | 655-661 |  | (A)6 | 286-291 |
|  | (T)6 | 783-788 |  | (T)6 | 378-383 |
|  | (G)6 | 1402-1407 |  | (A)6 | 2354-2359 |
|  | (A)6 | 1556-1561 |  | (A)6 | 2385-2390 |
|  |  |  |  | (A)6 | 2467-2472 |
|  |  |  |  | (A)6 | 593-598 |
|  |  |  |  | (A)6 | 808-813 |
|  |  |  |  | (T)6 | 955-960 |
|  |  |  |  | (A)6 | 1366-1371 |
| *Streptococcus pneumoniae* R6 |  |  |  | (T)6 | 69-74 |
|  |  |  |  | (T)6 | 378-383 |
|  |  |  |  | (GAA)3 | 544-552 |
|  |  |  |  | (TCC)3 | 2373-2381 |
| *Streptococcus pyogenes* MGAS5005 | (T)6 | 494-454 |  | (A)8 | 49-56 |
|  | (A)7 | 655-661 |  | (T)6 | 69-74 |
|  | (T)6 | 1111-1116 |  | (GAA)3 | 541-549 |
|  | (A)6 | 1138-1144 |  | (A)6 | 808-813 |
|  | (A)6 | 1308-1313 |  | (A)6 | 1366-1371 |
|  | (AAG)3 | 1673-1681 |  | (T)6 | 1425-1430 |
|  | (A)6 | 1922-1927 |  | (A)6 | 1446-1451 |
| *Vibrio cholerae* El Tor | (G)6 | 144-149 |  | (A)6 | 1506-1511 |
|  | (A)6 | 491-496 |  |  |  |
|  | (A)6 | 1282-1287 |  |  |  |
|  | (A)6 | 1820-1825 |  |  |  |
|  | (AAG)3 | 350-358 |  |  |  |

**Table S3. Primers used in this study**

| Reaction | Primer name | Primer sequences (5' to 3') |
| --- | --- | --- |
| Detection of *mutL* or 6bpΔ*mutL* | L-F1 | ATATCGACATCGAGCGTGGCGGCG |
|  | L-R1 | GCTTCCAGATCGTCAAGCGAGGCG |
| Construction of *mutL^Locked-1^* | LL1-F1 | GGAATTCCGCAGCGGGTAGGGTATGA^a^ |
|  | LL1-R1 | AGGGCAAGCTCCTCTTTTTT |
|  | LL1-F2 | AAGAGGAGCTTGCCCTGGCG |
|  | LL1-R2 | CGGATCCTCGCCAGTGCCTGTTCGA^b^ |
| Construction of *mutL^Locked-2^* | LL2-F1 | GGAATTCCAGCGGGTAGGGTATGATGT |
|  | LL2-R1 | CGCCAGCTCCTCTTTCTTAATGCCG |
|  | LL2-F2 | TGCGGCATTAAGAAAGAGGAGCTGG |
|  | LL2-R2 | CGGATCCGCCAGTGCCTGTTCGAGAAA |
| Construction of *mutL^Locked-3^* | LL3-F1 | GGAATTCATTTTGGTGGAGACGGGCTT |
|  | LL3-R1 | TAAATTCCGTCTTCTCGGTACGCAT |
|  | LL3-F2 | TCATGCGTACCGAGAAGACGGAATT |
|  | LL3-R2 | CGGATCCACTTCGTGCTTGGCGGGATG |
| Construction of *mutL^Locked-4^* | LL4-F1 | GGAATTCCGTCATGCCACCAGTAAAAT |
|  | LL4-R1 | GTTAACGCCGTAGTCGTGTGATTCG |
|  | LL4-F2 | CCGAATCACACGACTACGGCGTTAA |
|  | LL4-R2 | CGGATCCTAAAAGCGTGCGATAAACCT |
| Construction of 6bpΔ*mutL* | DL-F1 | GGAATTCCGCAGCGGGTAGGGTATGA |
|  | DL-R2 | CGGATCCTCGCCAGTGCCTGTTCGA |
| Construction of 6bpΔ*mutL^Locked^* | DL-F1 | GGAATTCCGCAGCGGGTAGGGTATGA |
|  | DL-R1 | GGCCAGCGCAAGCTCCTCTT |
|  | DL-F2 | AAGAGGAGCTTGCGCTGGCC |
|  | DL-R2 | CGGATCCTCGCCAGTGCCTGTTCGA |
| Construction of *mutS^Locked^* | SL-F1 | GGAATTCGAAATGGCGTTAATAGAGGG |
|  | SL-R1A | CCATTTTCTTACGCAACGCCTGTAC |
|  | SL-F2A | CAGGCGTTGCGTAAGAAAATGGGCG |
|  | SL-R2 | CGGATCCGGTAAATGTCGGGCAGGTGT |
| Construction of *mutL^L1-UL^* | L1L-F1 | GGAATTCCGCAGCGGGTAGGGTATGA |
|  | L1L-R1 | AGCGCCAGCTCCTCTTTTTT |
|  | L1L-F2 | AAGAGGAGCTGGCGCTGGCG |
|  | L1L-R2 | CGGATCCTCGCCAGTGCCTGTTCGA |
| Construction of *mutL^L0^* | L0-F1 | GGAATTCGGTATGATGTGGCGACGAAC |
|  | L0-R1 | TTCTCTACAAGCTCTTTAACAACCG |
|  | L0-F2 | TGTTAAAGAGCTTGTAGAGA |
|  | L0-R2 | CGGATCCAATGCAATACGACGGATGAT |

a. An EcoRI site is underlined; b. An BamHI site is underlined
